# Classifier Subset Selection for the Stacked Generalization Method Applied to Emotion Recognition in Speech

**DOI:** 10.3390/s16010021

**Published:** 2015-12-25

**Authors:** Aitor Álvarez, Basilio Sierra, Andoni Arruti, Juan-Miguel López-Gil, Nestor Garay-Vitoria

**Affiliations:** 1 Vicomtech-IK4. Human Speech and Language Technologies Department, Paseo Mikeletegi 57, Parque Científico y Tecnológico de Gipuzkoa, 20009 Donostia-San Sebastián, Spain; 2 University of the Basque Country (UPV/EHU), Paseo de Manuel Lardizabal 1, 20018 Donostia-San Sebastián, Spain; b.sierra@ehu.eus (B.S.); andoni.arruti@ehu.eus (A.A.); juanmiguel.lopez@ehu.eus (J.-M.L.-G.); nestor.garay@ehu.eus (N.G.-V.)

**Keywords:** affective computing, machine learning, speech emotion recognition

## Abstract

In this paper, a new supervised classification paradigm, called classifier subset selection for stacked generalization (CSS stacking), is presented to deal with speech emotion recognition. The new approach consists of an improvement of a bi-level multi-classifier system known as stacking generalization by means of an integration of an estimation of distribution algorithm (EDA) in the first layer to select the optimal subset from the standard base classifiers. The good performance of the proposed new paradigm was demonstrated over different configurations and datasets. First, several CSS stacking classifiers were constructed on the RekEmozio dataset, using some specific standard base classifiers and a total of 123 spectral, quality and prosodic features computed using in-house feature extraction algorithms. These initial CSS stacking classifiers were compared to other multi-classifier systems and the employed standard classifiers built on the same set of speech features. Then, new CSS stacking classifiers were built on RekEmozio using a different set of both acoustic parameters (extended version of the Geneva Minimalistic Acoustic Parameter Set (eGeMAPS)) and standard classifiers and employing the best meta-classifier of the initial experiments. The performance of these two CSS stacking classifiers was evaluated and compared. Finally, the new paradigm was tested on the well-known Berlin Emotional Speech database. We compared the performance of single, standard stacking and CSS stacking systems using the same parametrization of the second phase. All of the classifications were performed at the categorical level, including the six primary emotions plus the neutral one.

## 1. Introduction

Affective computing is an emerging area that tries to make human-computer interaction (HCI) more natural to humans. This area covers topics, such as affect or emotion recognition, understanding and synthesis. Computing systems can better adapt to human behavior taking non-verbal information into account. As Mehrabian suggested [1], verbal information comprises around 10% of the information transmitted between humans, while around 90% is non-verbal. This is why the inclusion of emotion-related knowledge in HCI applications improves the interaction by increasing the level of understanding and decreasing the ambiguity of the messages.

The expression of emotions by humans is multimodal [2]. Apart from verbal information (written or spoken text), emotions are expressed through speech [3,4,5], facial expressions [6], gestures [7] and other nonverbal clues (mainly psycho-physiological). With regard to the speech communication modality, the literature shows that several parameters (e.g., volume, pitch and speed) are appropriate to generate or recognize emotions [8]. This knowledge is important either to emulate diverse moods reflecting the user’s affective states or, in the case of a recognizer, to create patterns for classifying the emotions transmitted by the user.

Affective speech analysis refers to the analysis of spoken behavior as a marker of emotion, with a focus on the nonverbal aspects of speech [9]. Speech emotion recognition is particularly useful for applications that require natural human-machine interaction, in which the response to the user may depend on the detected emotion. Furthermore, it has been demonstrated that emotion recognition through speech can also be helpful in a wide range of other several scenarios, such as e-learning, in-card board safety systems, medical diagnostic tools, call centers for frustration detection, robotics, mobile communication or psychotherapy, among others.

Nevertheless, recognizing emotions from a human’s voice is a challenging task due to multiple issues. First, it must be considered that emotions’ expression is highly speaker, culture and language dependent. In addition, one spoken utterance can include more than one emotion, either as a combination of different underlying emotions in the same portion or as an individual expression of each emotion in different speech segments. Another interesting aspect is that there is no definitive consensus among the research community regarding which are the most useful speech features for emotion recognition. One possible cause may be the high impact of the variability introduced by the different speakers in commonly-used prosodic features. Finally, selecting the set of emotions to classify is an important decision, which can affect the performance of the speech emotion recognizer. Many works on the topic agree that any emotion is a combination of primary emotions. The primary six emotions include anger, disgust, fear, joy, sadness and surprise [10].

In this paper, we present a study on emotion recognition based on two different sets of speech features extracted from emotional audio signals recorded by professional actors. The analysis was performed on two datasets called RekEmozioand the Berlin Emotional Speech (Emo-DB) database. RekEmozio contains bilingual utterances in Basque and Spanish languages [11], whilst Emo-DB [12] includes sentences recorded in German. Both databases were designed to cover the six primary emotions plus the neutral one, and each recording contained one acted emotion. The classification approach was focused on the categorical recognition of the seven emotions included in the open Emo-DB and the RekEmozio dataset, which is currently in the process of becoming publicly available to the community.

The experiments were divided into three main phases. The first phase corresponded to the construction and evaluation of 10 base supervised classifiers, multi-classifier systems (bagging, boosting and standard stacking generalization) and bi-level multi-classifiers based on the classifier subset selection for stacked generalization (CSS stacking) method on the RekEmozio dataset. For this end, local and global speech parameters containing prosodic, quality and spectral information were computed from each recording through in-house feature extraction algorithms. The selected supervised classifiers for this phase were the following: Bayesian Network (BN), C4.5, k-Nearest Neighbors (kNN), KStar, Naive Bayes Tree (NBT), Naive Bayes (NB), One Rule (OneR), Repeated Incremental Pruning to Produce Error Reduction (RIPPER), Random Forest (RandomF) and Support Vector Machines (SVM). These classifiers were also used to build the CSS stacking classifiers in this first phase.

The aim of the second phase was to verify the efficiency of the CSS stacking classification paradigm on the RekEmozio dataset using: (1) a well-known set of acoustic parameters (extended version of the Geneva Minimalistic Acoustic Parameter Set (eGeMAPS)); and (2) different base classifiers in the first layer. For this purpose, CSS stacking classifiers were built using the best meta-classifier of the first phase. In the second phase, we applied the following base classifiers in the first layer: MultiLayer Perceptron (MLP), Radial Basis Function network (RBF), Logistic Regression (LR), C4.5, kNN, NB, OneR, RIPPER, RandomF and SVM. Hence, the MLP, RBF and LR classifiers were added with respect to the first phase, and the BN, KStar and NBT were discarded.

The third phase consisted of testing the CSS stacking paradigm over the well-known and open Emo-DB. To this end, the same standard classifiers and acoustic features (eGeMAPS) of the second phase were used to build single, standard stacking and CSS stacking classifiers. We decided to leave out the bagging and boosting classifiers because of their poor performance in the first phase.

The paper presents the results from three phases. Regarding the first phase, the results obtained when applying each classification method to each actor were presented, providing a comparison and discussion between each of the several classification paradigms proposed. Concerning the second phase, the results obtained with the CSS stacking classifier are given for each actor, and a comparison with the CSS stacking classifiers from the first phase is also provided. Finally, in the third phase, only the three classifiers with a better score have been presented for each of the constructed systems (single, standard stacking and CSS stacking). The performances of these classifier systems have been compared to each other and to other results obtained in related works in the literature over the same Emo-DB.

In addition, this paper aims to serve as a forum to announce that the RekEmozio dataset will be publicly available soon for research purposes. The aim is to provide the scientific community a new resource to make experiments in the speech emotion recognition field over audio and video acted recordings, several made by actors, others by amateurs, in the Spanish and Basque languages.

The rest of the paper is structured as follows. Section 2 introduces related work. Section 3 details the RekEmozio and Emo-DB datasets, in addition to the two sets of speech features used in this work. In Section 4, how EDA was applied for the stacking classification method is explained. Section 5 describes how the experiments that have been carried out were performed, specifying which techniques have been used in each step of the process. Section 6 explains the obtained results and provides a discussion. Section 7 concludes the paper and presents future work.

## 2. Related Work

Many studies in psychology have examined vocal expressions of emotions. Eyben *et al.* [8], Schuller *et al.* [3,9], Scherer [4] and Scherer *et al.* [5] provide reviews of these works. Besides, during recent years, the field of emotional content analysis of speech signals has been gaining growing attention. Scherer [4] described the state of research on emotion effects on voice and speech and discussed issues for future research efforts. The analyses performed by Sundberg *et al.* [13] suggested that the emotional samples could be better described by three physiological mechanisms, namely the parameters that quantified subglottal pressure, glottal adduction and vocal fold length and tension. Ntalampiras and Fakotakis [14] presented a framework for speech emotion recognition based on feature sets from diverse domains, as well as on modeling their evolution in time. Wu *et al.* [15] proposed modulation spectral features (MSFs) for the automatic recognition of human affective information from speech. More recently, [16] proposed a novel feature extraction based on multi-resolution texture image information (MRTII), including a BS-entropy-based acoustic activity detection (AAD) module and using an SVM classifier. They improve the performance of other systems based on Mel-frequency cepstral coefficients (MFCC), prosodic and low-level descriptor (LLD) features for three artificial corpora (Emo-DB, eNTERFACE, KHUSC-EmoDB) and a mixed database. There have been several challenges on emotion and paralinguistics in INTERSPEECH, as shown in [3,9].

An important issue to be considered in the evaluation of an emotional speech recognizer is the quality of the data used to assess its performance. The proper design of emotional speech databases is critical to the classification task. Work in this area has made use of material that was recorded during naturally-occurring emotional states of various sorts, that recorded speech samples of experimentally-induced specific emotional states in groups of speakers and that recorded professional or lay actors asked to produce vocal expressions of emotion as based on emotion labels and/or typical scenarios [4].

Several reviews on emotional speech databases have been published. Douglas-Cowie *et al.* [17] provided a list of 19 data collections, while El Ayadi *et al.* [18] and Ververidis and Kotropoulos [19] provided a record of an overview of 17 and 64 emotional speech data collections, respectively. Most of these references of affective databases are related to English, while fewer resources have been developed for other languages. This is particularly true to languages with a relatively low number of speakers, such as the Basque language. To the authors’ knowledge, the first affective database in Basque is the one presented by Navas *et al.* [20]. Concerning Spanish, the work of Iriondo *et al.* [21] stands out; and relating to Mexican Spanish, the work of Caballero-Morales [22] can be highlighted. On the other hand, the RekEmozio dataset is a multimodal bilingual database for Spanish and Basque [11], which also stores information that came from processes of some global speech feature extractions for each audio recording.

Popular classification models used for emotional speech classification include, among others, different decision trees [23], SVM [8,24,25,26], neural networks [27] and hidden Markov models (HMM) [28,29]. Which one is the best classifier often depends on the application and corpus [30]. El Ayadi *et al.*[18] and Ververidis and Kotropoulos [19] provide a review of appropriate techniques in order to classify speech into emotional states.

In order to combine the benefits of different classifiers, classifier fusion is starting to become common, and several different examples can be found in the literature [31]. Pfister and Robinson [30] proposed an emotion classification framework that consists of n(n-1)/2 pairwise SVMs for n labels, each with a differing set of features selected by the correlation-based feature selection algorithm. Arruti *et al.* [32] used four machine learning paradigms (IB, ID3, C4.5, NB) and evolutionary algorithms to select feature subsets that noticeably optimize the automatic emotion recognition success rate. Schuller *et al.* [24] combined SVMs, decision trees and Bayesian classifiers to yield higher classification accuracy. Scherer *et al.* [33] combined three different KNN classifiers to improve the results. Chen *et al.* [34] proposed a three-level speech emotion recognition model combining Fisher rate, SVM and artificial NN in comparative experiments. Attabi and Dumouchel [35] proved that, in the context of highly unbalanced data classes, back-end systems, such as SVMs or a multilayer perceptron (MLP), can improve the emotion recognition performance achieved by using generative models, such as Gaussian mixture models (GMMs), as front-end systems, provided that an appropriate sampling or importance weighting technique is applied. Morrison *et al.* [36] explored two classification methods that had not previously been applied in affective recognition in speech: stacked generalization and unweighted vote. They showed how these techniques can yield an improvement over traditional classification methods. Huang *et al.* [37] developed an emotion recognition system for a robot pet using stacked generalization ensemble neural networks as the classifier for determining human affective state in the speech signal. Wu and Liang [38] presented an approach to emotion recognition of affective speech based on multi-classifiers using acoustic-prosodic information (AP) and semantic labels. Three types of models, GMMs, SVMs and MLPs, are adopted as the base-level classifiers. A meta decision tree (MDT) is then employed for classifier fusion to obtain the AP-based emotion recognition confidence. Several methods have been used for decision fusion in speech emotion recognition. Kuang and Li [39] proposed the Dempster–Shafer evidence theory to execute decision fusion among the three kinds of emotion classifiers to improve the accuracy of the speech emotion recognition. Huang *et al.* [40] used FoCalfusion, AdaBoost fusion and simple fusion on their studies of the effects of acoustic features, speaker normalization methods and statistical modeling techniques on speaker state classification.

## 3. Case Study

In this section, the main characteristics of the RekEmozio and Emo-DB datasets used for the experiments are presented first. In addition, the speech features used to train and test classifiers are described.

### 3.1. RekEmozio Dataset

The RekEmozio dataset was created with the aim of serving as an information repository to perform research on user emotions. The RekEmozio dataset is based on data acquired through user interaction and metadata used to describe and label each interaction and provides access to the data stored and the faculty of performing transactions over them, so new information can be added to the dataset by analyzing the data included in it. When building the RekEmozio dataset, the aim was adding descriptive information about the performed recordings, so processes, such as extracting speech parameters and video features, may be done currently on them.

The RekEmozio dataset is composed of audio and video acted recordings, several made by professional actors, while others are by amateurs. In this study, we use the audio recordings made by professional actors. Those recordings are either in the Basque or Spanish languages.

The classification of emotions was performed at the categorical level. For this purpose, seven emotions were used: the six basic emotions described by [6], that is sadness, fear, joy, anger, surprise and disgust, and a neutral emotion. The selection of these specific emotions was based on the work by Ekman and Friesen [6], which suggested that these emotions are universal for all cultures. This is interesting considering the bilingualism of the RekEmozio dataset.

There are 88 different sentences with 154 recordings over them for each actor. Seven actors recorded sentences for Basque, while 10 recorded for Spanish. The total length of the audio recordings was 130${}^{\prime }$41${}^{\prime }$${}^{\prime }$ for Basque and 166${}^{\prime }$17${}^{\prime }$${}^{\prime }$ for Spanish.

A validation for normative study was performed by experimental subjects in order to obtain affective values for each recording and to see what the validity of the recorded material and the affective values for each recording are [41]. Achieved results show that the material recorded in the RekEmozio database was correctly identified by 57 experimental subjects, with a mean accuracy of 66.5% for audio recordings. In Table 1, audio recognition accuracy percentages for the different types of utterances (depending on the language) are presented. It has also to be noted that several automatic emotion recognition systems have used the RekEmozio dataset in previous works, such as [32,42].

**Table 1:** Human recognition accuracy percentages for utterances as a function of language and emotions (taken from [41]).

	Sadness	Fear	Joy	Anger	Surprise	Disgust	Neutral
**Spanish**	75%	51%	78%	71%	66%	52%	80%
**Basque**	77%	52%	68%	74%	59%	51%	77%

The RekEmozio dataset is currently in the process of being made publicly available (until the process is completed and as the RekEmozio dataset remains unavailable from a public repository, anyone interested can contact Karmele López de Ipiña or the co-author Nestor Garay-Vitoria with the aim of the community having access to the dataset for research purposes.

A complete description of the RekEmozio dataset characteristics can be seen in [11].

### 3.2. Emo-DB

The widely extended German Emo-DB [12] is composed of recordings of 10 actors (five female and five male), which simulated the six primary emotions defined by [6] plus the neutral one. The complete database was evaluated through a perception test with 20 subjects, achieving a human performance of 84% accuracy [43]. The Emo-DB is publicy available via the Internet.

### 3.3. Speech Features

The selection of suitable features to extract from the voice signal is one of the most difficult and important decisions to be made in the speech emotion recognition task. It is even more critical when pattern recognition techniques are involved, since they are highly dependent on the domain and training material. The voice characteristics most commonly employed in the literature involve the computation of prosodic and continuous features, qualitative features, spectral features and Teager energy operator (TEO)-based features. A deep description of these categories is given in the survey on speech emotion recognition presented in [18]. With the aim of creating a common baseline and agreed set of speech features to use by the speech emotion recognition community, a minimalistic set of voice parameters were recently compiled and presented in [8].

The feature extraction method is also a regular topic of discussion within the speech emotion recognition field. Because of the non-stationary nature of speech signals, the features are usually extracted from overlapped small frames, which consist of a few milliseconds portions of signal. The features extracted at the frame level are known as local features. Using these local features and computing statistics among them, global features are also usually calculated at the utterance level. Even if the best results were obtained in many works [44,45,46] using global features instead of local features, it is not clear whether global features performed better for any emotion classification. In fact, in the work presented in [28], they proved that global features do not perform correctly when recognizing emotions with similar arousal, e.g., happiness and anger.

In this work, two sets of speech features were computed along the three phases. In the first phase, local and global features containing prosodic, spectral and quality information were extracted using in-house algorithms, considering a total set of 123 features for each spoken utterance. The extraction of local features was done at both the frame and region levels. In the first case, a 20-millisecond frame-based analysis window was used, with an overlapping of 10 milliseconds. Concerning the feature extraction at the region level, the work presented in Tato *et al.* [47] was followed. They defined a technique for signal treatment and information extraction from emotional speech, not only extracting information by frames, but also by regions consisting of more than three consecutive speech frames. With regard to global features, statistics containing measures, such as the mean, variance, standard deviation and the maximum and minimum values and their positions, were computed, among others. The full set of the 123 features we used in the first phase of this work, including local characteristics and their correlated global statistics, were described in more detail in [32].

With regard to the second and third phases, the extended version (eGeMAPS) of the Geneva Minimalistic Acoustic Parameter Set (GeMAPS) was used to extract a different set of speech features. The complete description of the parameters involved in the eGeMAPS set is given in [8]. The extraction of this set of features was done through the OpenSMILE toolkit presented in [48].

## 4. Classifier Subset Selection to Improve the Stacked Generalization Method

One of the main goals of this work was the construction of a multi-classifier system with optimal selection of the base classifiers in the speech emotion recognition domain. For this purpose, a method proposed in [49] was applied to select an optimal classifier subset by means of the estimation of distribution algorithms (EDAs).

In order to combine the results of the base classifiers, we employed stacked generalization (SG) as a multi-classifier system. Stacked generalization is a well-known ensemble approach, and it is also called stacking [50,51]. While ensemble strategies, such as bagging or boosting, obtain the final decision after a vote among the predictions of the individual classifiers, SG applies another individual classifier to the predictions in order to detect patterns and improve the performance of the vote.

**Figure 1:**
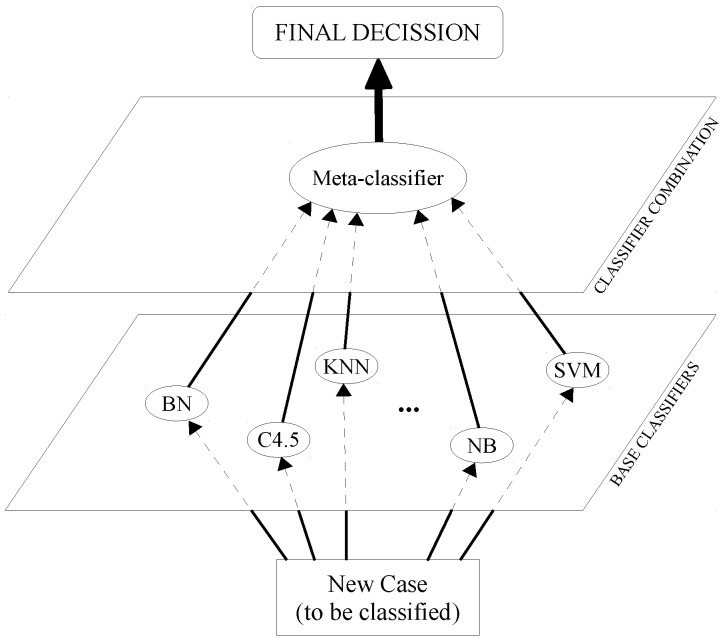
Stacked generalization schemata.

As can be seen in Figure 1, SG is divided into two levels: for Level 0, each individual classifier makes a prediction independently, and for Level 1, these predictions are treated as the input values of another classifier, known as the meta-classifier, which returns the final decision.

The data for training the meta-classifier is obtained after a validation process, where the outputs of the Level 0 classifiers are taken as attributes, and the class is the real class of the example. This implies that a new dataset is created in which the number of predictor variables corresponds to the number of classifiers of the bottom layer, and all of the variables have the same value range as the class variable.

Within this approach, using many classifiers can be very effective, but selecting a subset of them can reduce the computational cost and improve the accuracy, assuming that the selected classifiers are diverse and independent. It is worth mentioning that a set of accurate and diverse classifiers is needed in order to be able to improve the classification results obtained by each of the individual classifiers that are to be combined. This fact has been taken into account to select the classifiers that take part in the first layer of the stacked generalization multi-classifier used.

**Figure 2:**
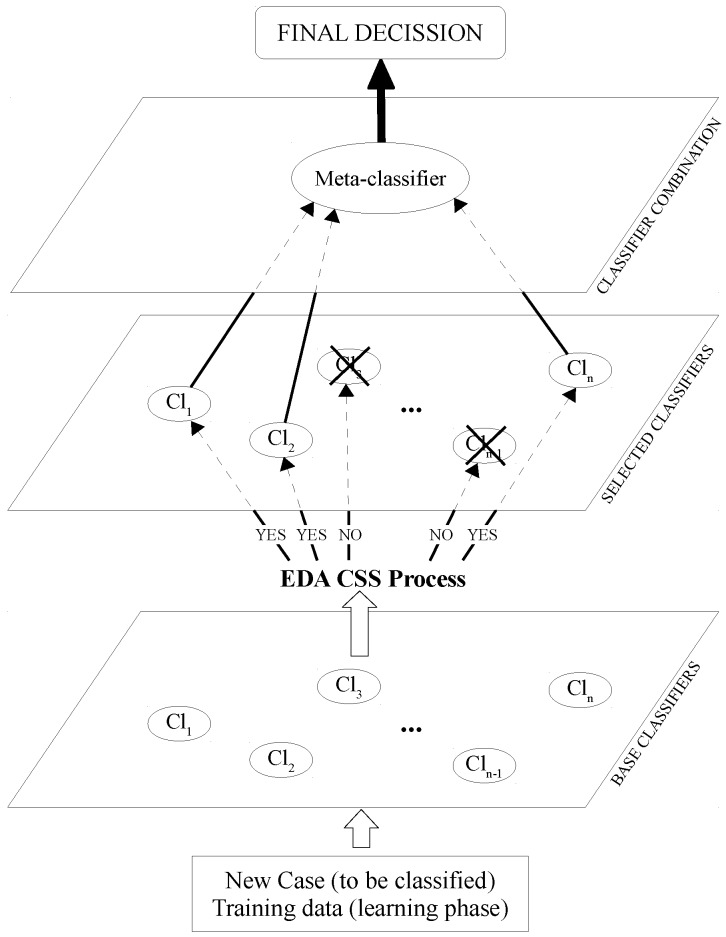
Classifier subset selection stacked generalization.

In [49], an extension of the staking generalization approach is proposed, reducing the number of classifiers to be used in the final model. This new approach is called classifier subset selection (CSS), and a graphical example is illustrated in Figure 2. As can be seen, an intermediate phase is added to the multi-classifier to select a subset of Level 0 classifiers. The classification accuracy is the main criterion to make this selection. As can be seen in Figure 2, discarded classifiers, those with an X, are not used in the multi-classifier.

The method used to select the classifiers could be any, but in this type of scenario, evolutionary approaches are often used. Currently, some of the best known evolutionary algorithms for feature subset selection (FSS) are based on EDAs [52]. EDA combines statistical learning with population-based search in order to automatically identify and exploit certain structural properties of optimization problems. Inza *et al.* [53] proposed an approach that used an EDA called the estimation of Bayesian network algorithm (EBNA) [54] for an FSS problem. Seeing that in [55], EBNA shows better behavior than genetic and sequential search algorithms for FSS problems (and hence, for CSS in this approach), we decided to use EBNA. Moreover, EBNA has been selected as the model in the recent work that analyses the behavior of the EDAs [56].

**Figure 3:**
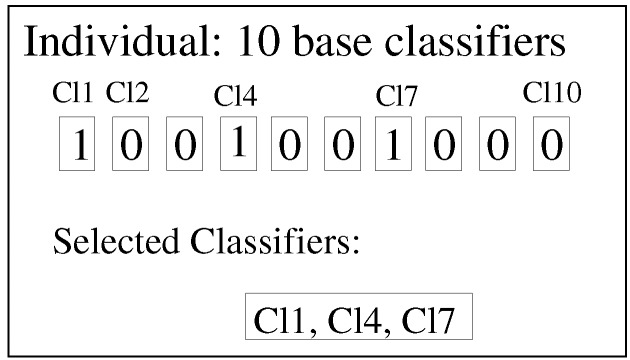
The combinations of base classifiers as the estimation of distribution algorithm (EDA) individuals.

In our approach, an individual in the EDA algorithm is defined as an *n*-tuple with 0,1 binary values, so-called binary encoding. Each position in the tuple refers to a concrete base classifier, and the value indicates whether this classifier is used (1 value) or not (0 value). An example with 10 classifiers (the value used in this paper) can be seen in Figure 3. In this example, Classifiers 1, 4 and 7 (Cl1, Cl4 and Cl7) are the selected classifiers, and the remaining seven are not used.

Once an individual has been sampled, it has to be evaluated. The aim is to consider the predictive power of each subset of base classifiers. To this end, a multi-classifier is built for each individual using the corresponding subset of classifiers, and the obtained validated accuracy is used as the fitness function. Thus, when looking for the individual that maximizes the fitness function, the EDA algorithm is also searching the optimal subset of base classifiers.

## 5. Experiments

In this section, the whole experimental design is described. Firstly, the single classifiers employed in all phases are presented, followed by the definition of the experiment steps. In the end, the experimental setup and the main measure used for the analysis of the obtained results are detailed.

### 5.1. Base Classifiers

#### 5.1.1. First Phase

The experiments of the first phase were carried out over 10 well-known machine-learning (ML) supervised classification algorithms through the Weka software package [57], which includes a collection of machine learning algorithms for data mining tasks. A brief description of the classifiers of the first phase is presented below.

- Bayesian Networks (BN): A Bayesian network [58], belief network or directed acyclic graphical model is a probabilistic graphical model that represents a set of random variables and their conditional independencies via a directed acyclic graph (DAG).
- C4.5: C4.5 [59] represents a classification model by a decision tree. The tree is constructed in a top-down way, dividing the training set and beginning with the selection of the best variable in the root of the tree.
- k-Nearest Neighbors (KNN): This algorithm is a case-based, nearest-neighbor classifier [60]. To classify a new test sample, a simple distance measure is used to find the training instance closest to the given test instance, and then, it predicts the same class as this nearest training instance.
- KStar: This classifier is an instance-based algorithm that uses an entropy-based distance function [61].
- Naive Bayes Tree (NBT): This classification method uses a decision tree with naive Bayes classifiers at the leaves [62].
- Naive Bayes (NB): The naive Bayes rule [63] uses the Bayes theorem to predict the class for each case, assuming that the predictive genes are independent given the category. To classify a new sample characterized by *d* genes $X=(X_{1},X_{2},...,X_{d})$, the NB classifier applies the following rule:
  $$c_{NB}=\arg\max_{c_{j}\in C}p\left(c_{j}\right)\prod_{i=1}^{d}p\left(x_{i}|c_{j}\right)$$
  where $c_{NB}$ denotes the class label predicted by the naive Bayes classifier, and the possible classes of the problem are grouped in $C=\{c_{1},\ldots ,c_{l}\}$.
- One Rule (OneR): This simple classification algorithm is a one-level decision tree, which tests just one attribute [64]. The chosen attribute is the one that produces the minimum error.
- Repeated Incremental Pruning to Produce Error Reduction (RIPPER): The rule-based learner presented in [65] forms rules through a process of repeated growing (to fit training data) and pruning (to avoid overfitting). RIPPER handles multiple classes by ordering them from least to most prevalent and then treating each in order as a distinct two-class problem.
- Random Forest (RandomF): This constructs a combination of many unpruned decision trees [66]. The output class is the mode of the classes output by individual trees.
- Support Vector Machines (SVM): These are a set of related supervised learning methods used for classification and regression [67]. Viewing input data as two sets of vectors in a *n*-dimensional space, an SVM will construct a separating hyperplane in that space, one that maximizes the margin between the two datasets.

#### 5.1.2. Second and Third Phases

For the second and third phases, the BN, K-Star and NBT classifiers of the first phase were discarded, the rest of the base classifiers were kept, and three new classifiers were included for experimentation, including multilayer perceptron, radial basis function networks and logistic regression, as they are described below.

- Multilayer Perceptron (MLP): A multilayer perceptron is a feedforward artificial neural network model to map sets of input data onto a set of appropriate outputs [68]. An MLP consists of multiple layers of nodes in a directed graph, with each layer fully connected to the next one. Except for the input nodes, each node is a processing element with a nonlinear activation function.
- Radial Basis Function (RBF) network: A radial basis function network is an artificial neural network using radial basis functions as activation functions [69]. The output of the network is a linear combination of radial basis functions of the inputs and neuron parameters.
- Logistic Regression: A logistic regression (also known as logit regression or logit model ) [70] is considered in statistics a regression model where the dependent variable is categorical.

As it can be seen from the three phases, classifiers with different approaches for learning and widely used in different classification tasks were selected. The goal was to combine them in a multi-classifier to maximize the benefits of each modality by intelligently fusing their information and by overcoming the limitations of each modality alone.

### 5.2. Experimental Steps

As described above, the experiments were organized in three phases. In the first phase, single classifiers, standard multi-classifier systems and CSS stacking classifiers were built over the RekEmozio dataset and compared. During the second phase, new CSS stacking classifiers were built for each of the 17 actors in the same dataset, using new parametrization and configuration of the base classifiers in the first layer. These CSS stacking systems were compared to the CSS stacking classifiers of the first phase. Finally, new single, standard stacking and CSS stacking classifiers were built on the Emo-DB, employing the same acoustic features and standard classifiers of the second phase.

#### 5.2.1. First Phase

1. Single classifiers: build 10 classifiers on the RekEmozio dataset, applying the 10 base machine learning algorithms of the first phase to the training dataset and get validated classification accuracies.
2. Standard multi-classifiers: build one classifier on the RekEmozio dataset applying bagging, another one applying boosting and ten more applying stacking generalization, one for each base classifier at Level 1, and get validated classification accuracies.
3. Classifier subset selection for stacked generalization: build 10 stacking generalization classifiers on the RekEmozio dataset, one for each base classifier acting as a meta-classifier at Level 1, and select, by means of an evolutionary algorithm, a subset of the ten classifiers to participate in the Level 0 layer.

It is worth mentioning that in all of the experiments, a 10-fold cross-validation technique was used. In the case of the classifier subset selection method, this validation was also employed to select the classifier configuration that performed better on average.

#### 5.2.2. Second Phase

1. Classifier subset selection for stacked generalization: build one stacking generalization classifier on the RekEmozio dataset, using the best meta-classifiers from the first phase, and select, by means of an evolutionary algorithm, a subset of the ten classifiers to participate in the Level 0 layer.

#### 5.2.3. Third Phase

1. Single classifiers: build 10 classifiers on the Emo-DB, applying the 10 base machine learning algorithms of the second phase to the training dataset, and get validated classification accuracies.
2. Standard multi-classifiers: build ten classifiers applying stacking generalization, one for each base classifier at Level 1, and get validated classification accuracies.
3. Classifier subset selection for stacked generalization: build 10 stacking generalization classifiers on the Emo-DB, one for each base classifier acting as the meta-classifier at Level 1, and select, by means of an evolutionary algorithm, a subset of the classifiers to participate in the Level 0 layer.

### 5.3. Experimental Setup

In all of the experiments, 10-fold cross-validation [71] was applied to get a validated classification accuracy (well-classified rate), and this accuracy has been the criterion to define the fitness of an individual, inside the evolutionary algorithm.

For classifier subset selection, the selected EDA algorithm was EBNA, with Algorithm B [72] for structural learning of the Bayesian network. Population size *N* was set to 50 individuals, representing 50 combinations of classifiers; the number *S* of selected individuals at each generation was 20 (40% of the population size); and the maximum number of generations of new individuals was set to 10.

### 5.4. Obtained Results Analysis

The main measure that has been used in this study to evaluate classification methods was the accuracy. The accuracy reflects how many times the emotions are recognized, comparing this to the metadata stored in the RekEmozio and Emo-DB datasets. Accuracy is expressed as a percentage with respect to the total of the recordings.

## 6. Results and Discussion

### 6.1. First Phase

Table 2 presents the results obtained for each of the 17 actors in the first phase when a single classification is applied for categorical emotion recognition, in addition to the mean values and standard deviation (SD) of each classifier in the last two rows. The best accuracies obtained per actor are highlighted in bold. The results suggest that SVM is the classifier that performs better when the single classifier method is applied, as for 13 of the 17 actors, the SVM classifier obtains the best results compared to the rest of the single classifiers, and its mean value is 6.43 percentage points higher than the second mean value. Only BN and RandomF get the better accuracies than SVM in the single classification, in the case of two actors for each one. The best accuracy (73.79%) is achieved for the actor P1. The rest of best accuracies for each actor range from 41.82% (P8) to 68.93% (P6).

**Table 2:** First phase. Accuracy percentages for each person using single classifiers. Mean and SD rows denote the average and standard deviation for each classifier considering all of the actors. BN, Bayesian network; NBT, naive Bayes tree; OneR, one rule; RIPPER, repeated incremental pruning to produce error reduction; RandomF, random forest.

	BN	C4.5	KNN	KStar	NBT	NB	OneR	RIPPER	RandomF	SVM
**P1**	69.90%	64.08%	65.05%	54.37%	55.34%	61.17%	53.40%	48.54%	64.08%	**73.79%**
**P2**	58.25%	45.63%	49.51%	36.89%	44.66%	39.81%	34.95%	46.60%	53.40%	**66.02%**
**P3**	46.60%	45.63%	49.51%	36.89%	44.66%	39.81%	34.95%	46.60%	34.95%	**53.40%**
**P4**	**59.22%**	43.69%	39.81%	35.92%	43.69%	34.95%	45.63%	33.01%	54.37%	**59.22%**
**P5**	52.43%	42.72%	41.75%	36.89%	54.37%	49.51%	42.72%	48.54%	52.43%	**68.93%**
**P6**	53.40%	48.54%	46.60%	38.83%	56.31%	38.83%	43.69%	46.60%	63.11%	**66.99%**
**P7**	42.72%	33.98%	32.04%	25.24%	41.75%	37.86%	38.83%	38.83%	**50.49%**	45.63%
**P8**	18.18%	29.09%	29.09%	20.91%	29.09%	19.09%	12.73%	12.73%	26.36%	**41.82%**
**P9**	44.55%	43.64%	37.27%	33.64%	40.00%	38.18%	30.00%	36.36%	43.64%	**52.73%**
**P10**	54.55%	43.64%	50.91%	26.36%	52.73%	55.45%	30.00%	41.82%	58.18%	**64.55%**
**P11**	56.36%	42.73%	37.27%	28.18%	50.00%	43.64%	38.18%	42.73%	55.45%	54.55%
**P12**	44.55%	32.73%	37.27%	27.27%	31.82%	27.27%	38.18%	39.09%	37.27%	45.45%
**P13**	44.55%	40.91%	33.64%	35.45%	44.55%	27.27%	45.45%	42.73%	50.91%	**61.82%**
**P14**	**64.55%**	51.82%	37.27%	31.82%	54.55%	35.45%	40.00%	45.45%	60.00%	56.36%
**P15**	51.82%	53.64%	53.64%	37.27%	52.73%	40.91%	40.00%	57.27%	**62.73%**	**62.73%**
**P16**	58.18%	48.18%	47.27%	36.36%	50.00%	40.91%	43.64%	54.55%	53.64%	**59.09%**
**P17**	50.91%	46.36%	40.91%	23.64%	45.45%	37.27%	40.00%	40.91%	**53.64%**	50.91%
***Mean***	51.22%	44.53%	42.87%	33.29%	46.57%	39.26%	38.37%	42.49%	51.45%	**57.88%**
***SD***	10.98	7.91	8.83	7.57	7.69	9.72	8.52	9.51	10.13	8.69

**Table 3:** First phase. Accuracy percentages for each person using stacking and bagging and boosting multi-classifiers. Mean and SD rows denote the average and standard deviation for each standard multi-classifier considering all of the actors.

	BN	C4.5	KNN	KStar	NBT	NB	OneR	RIPPER	RandomF	SVM	Bagging	Boosting
**P1**	64.08%	59.22%	72.82%	57.28%	67.96%	61.17%	43.69%	62.14%	66.99%	**73.79%**	65.05%	34.95%
**P2**	47.57%	54.37%	42.72%	36.89%	**60.19%**	47.57%	36.89%	49.51%	49.51%	52.43%	57.28%	30.10%
**P3**	49.51%	44.66%	48.54%	41.75%	46.60%	**58.25%**	45.63%	46.60%	52.43%	49.51%	49.51%	35.92%
**P4**	49.51%	48.54%	41.75%	33.98%	53.40%	45.63%	39.81%	42.72%	50.49%	44.66%	**55.34%**	33.01%
**P5**	51.46%	45.63%	48.54%	32.04%	**55.34%**	50.49%	41.75%	52.43%	**55.34%**	**55.34%**	54.37%	32.04%
**P6**	**59.22%**	56.31%	53.40%	42.72%	**59.22%**	54.37%	55.34%	54.37%	**59.22%**	55.34%	52.43%	32.04%
**P7**	46.60%	33.98%	41.75%	39.81%	46.60%	44.66%	37.86%	41.75%	46.60%	40.78%	**48.54%**	37.86%
**P8**	**31.82%**	23.64%	30.91%	24.55%	29.09%	25.45%	19.09%	20.91%	23.64%	24.55%	30.91%	17.27%
**P9**	45.45%	41.82%	47.27%	36.36%	49.09%	45.45%	41.82%	36.36%	**50.91%**	46.36%	**50.91%**	30.91%
**P10**	55.45%	55.45%	53.64%	40.91%	56.36%	48.18%	37.27%	52.73%	**60.91%**	55.45%	54.55%	35.45%
**P11**	49.09%	48.18%	43.64%	39.09%	49.09%	40.91%	39.09%	50.00%	55.45%	52.73%	**58.18%**	35.45%
**P12**	39.09%	35.45%	36.36%	20.91%	38.18%	48.18%	30.91%	35.45%	35.45%	43.64%	**47.27%**	35.45%
**P13**	39.09%	41.82%	34.55%	30.91%	42.73%	43.64%	40.91%	**46.36%**	40.91%	40.91%	44.55%	34.55%
**P14**	50.00%	56.36%	57.27%	40.91%	63.64%	62.73%	37.27%	56.36%	**60.00%**	**60.00%**	44.55%	34.55%
**P15**	44.55%	57.27%	50.91%	40.91%	57.27%	52.73%	37.27%	50.91%	56.36%	59.09%	**60.91%**	36.36%
**P16**	55.45%	45.45%	49.09%	40.91%	50.00%	49.09%	40.00%	48.18%	54.55%	48.18%	**56.36%**	32.73%
**P17**	41.82%	44.55%	38.18%	38.18%	46.36%	44.55%	36.36%	43.64%	43.64%	**50.00%**	43.64%	30.91%
***Mean***	48.22%	46.63%	46.55%	37.54%	51.24%	48.41%	38.88%	46.50%	50.73%	50.16%	**51.43%**	32.91%
***SD***	7.44	9.05	9.33	7.53	9.04	8.08	6.82	8.96	9.86	9.86	7.53	4.32

The performance of the standard multi-classifiers systems for all of the actors in the first phase is presented in Table 3, with mean and SD values in the last two rows. In the first 10 columns, the results obtained by the stacked generalization method with the single classifiers as meta classifiers are presented. In addition, the accuracy achieved by the bagging and boosting multi-classifiers are shown in the last two columns. The best results per actor are marked in bold. In contrast to single classifiers, there is no meta classifier that performs much better than the others. This is evident looking at their mean values, with four classifiers in the range from 50.16% to 51.43%, showing low differences between them. For seven actors, the best accuracies are reached using the bagging multi-classifier; RandomF gets the best accuracies for five actors, SVM for four actors, NBT for three actors, BN for two actors, and NB and RIPPER get the best accuracy for one actor each. This happens because in some cases, there are several meta-classifiers that get the best accuracies for a given actor (for example, P5). On the other hand, for 14 actors, the worst results are obtained with the boosting multi-classifier. Compared to the results from the single classifiers in Table 2, only for three of the 17 actors (P3, P11 and P12) are improvements achieved on their best classification results using multi-classifiers. For the rest of the actors, the accuracies are lower when compared to single classifiers.

The results reached by CSS stacking classifiers in the first phase are shown in Table 4, including their mean and SD values. If we focus on the highlighted values, which correspond to the best accuracies for each of the actors, the SVM classifier achieves the best scores, on average, when it is used as a meta classifier (an increase of 2.22 percentage points over the second one) and for 13 actors. The other actors obtained best accuracies with C4.5, NBT, NB and RIPPER meta-classifiers. In general, the best accuracies are improved using the CSS stacking classification method against the standard multi-classifiers. Besides, if we compared the results from CSS stacking with the best accuracies achieved by the single classifiers, 13 of the 17 actors obtain higher classification results. This point is clearly demonstrated in Table 5, where the best accuracies obtained per actor are presented for each of the classification methods, including single classifiers, multi-classifiers (boosting, bagging and stacking) and CSS stacking classifiers. In addition, two columns are presented that show the differences obtained when comparing the best accuracies achieved by multi-classifiers against the single classifiers (Differences_1), and the ones obtained by the CSS stacking classifiers against the best between the single and multi-classifiers (Differences_2).

**Table 4:** First phase. Accuracy percentages for each person applying CSS stacking with the EDA classification method. Mean and SD rows denote the average and standard deviation for each classifier working as meta classifiers and considering all of the actors.

	BN	C4.5	KNN	KStar	NBT	NB	OneR	RIPPER	RandomF	SVM
**P1**	71.84%	70.87%	**73.79%**	72.82%	68.93%	70.87%	44.66%	68.93%	71.84%	**73.79%**
**P2**	66.02%	70.87%	**73.79%**	72.82%	60.19%	70.87%	36.89%	68.93%	71.84%	**73.79%**
**P3**	57.28%	70.87%	**73.79%**	72.82%	65.05%	70.87%	45.63%	68.93%	71.84%	**73.79%**
**P4**	55.34%	60.19%	51.46%	53.40%	51.46%	**62.14%**	39.81%	51.46%	59.22%	61.17%
**P5**	55.34%	63.11%	57.28%	49.51%	51.46%	62.14%	42.72%	58.25%	62.14%	**65.05%**
**P6**	64.08%	**66.99%**	59.22%	58.25%	50.49%	54.37%	57.28%	60.19%	66.02%	59.22%
**P7**	51.46%	47.57%	51.46%	41.75%	39.81%	51.46%	37.86%	47.57%	50.49%	**52.43%**
**P8**	30.91%	34.55%	35.45%	30.00%	**43.64%**	34.55%	26.36%	33.64%	33.64%	32.73%
**P9**	48.18%	50.91%	48.18%	47.27%	46.36%	45.45%	42.73%	47.27%	50.00%	**54.55%**
**P10**	58.18%	60.91%	58.18%	56.36%	45.45%	60.91%	37.27%	**61.82%**	60.91%	60.91%
**P11**	53.64%	53.64%	54.55%	54.55%	46.36%	55.45%	41.82%	50.91%	56.36%	**60.00%**
**P12**	34.55%	49.09%	45.45%	42.73%	32.73%	49.09%	40.91%	41.82%	49.09%	**51.82%**
**P13**	47.27%	**59.09%**	51.82%	54.55%	52.73%	52.73%	40.91%	52.73%	50.91%	**59.09%**
**P14**	50.91%	62.73%	64.55%	59.09%	55.45%	64.55%	38.18%	64.55%	63.64%	**66.36%**
**P15**	58.18%	62.73%	59.09%	59.09%	50.00%	60.91%	37.27%	59.09%	59.09%	**63.64%**
**P16**	55.45%	50.91%	59.09%	53.64%	44.55%	60.00%	40.91%	52.73%	56.36%	**60.91%**
**P17**	48.18%	50.91%	52.73%	45.45%	45.45%	49.09%	37.27%	44.55%	48.18%	**54.55%**
***Mean***	53.34%	58.00%	57.05%	54.36%	50.01%	57.38%	40.50%	54.90%	57.74%	**60.22%**
***SD***	9.57	9.34	9.73	10.87	8.40	9.26	5.75	9.63	9.55	9.37

**Figure 4:**
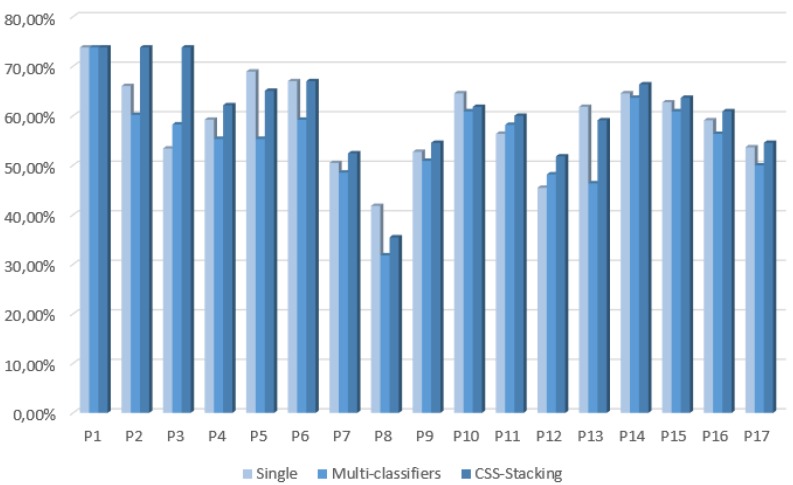
First phase. Best accuracies per person considering single, multi-classifiers and CSS stacking with EDA classification methods.

The results from Table 5 show that using multi-classifiers does not outperform the classification accuracies in this classification problem. Nevertheless, when applying the CSS stacking classification method, the improvements are noticeable for many of the actors. As is detailed in the last column Differences_2, 11 actors outperform the best accuracies when compared to the ones obtained with the single and multi-classifiers, giving a mean increase of 1.48 percentage points. The highest improvement is achieved by the actor P3, which increases the accuracy by 15.54 percentage points. The rest of the improvements are in the range from 0.91 to 7.77 points. In addition, two of the actors (P1 and P6) reached the same best accuracy with no significant improvements, and there are four cases (P5, P8, P10 and P13) where the single classifiers reach the best accuracies. A comparison of the best accuracies obtained per actor for each of the classification methods is presented in Figure 4.

**Table 5:** First phase. Best accuracy per person by using each classification method. Improvements comparing the best accuracy from multi-classifiers (bagging, boosting and stacking) against single classifiers are presented in the Differences_1 column. In addition, the improvements between the CSS stacking with EDA and the best accuracy from both single and standard multi-classifiers are shown in the Differences_2 column. Mean and SD rows denote the average and standard deviation for each classification method and the type of differences considering all of the actors. Differences are expressed in percentage points.

	Single	Bagging	Boosting	Stacking	Differences_1	CSS Stacking	Differences_2
**P1**	**73.79%**	65.05%	34.95%	**73.79%**	0.00	**73.79%**	0.00
**P2**	66.02%	57.28%	30.10%	60.19%	−5.83	**73.79%**	+7.77
**P3**	53.40%	49.51%	35.92%	58.25%	+4.85	**73.79%**	+15.54
**P4**	59.22%	55.34%	33.01%	53.40%	−3.88	**62.14%**	+2.92
**P5**	**68.93%**	54.37%	32.04%	55.34%	−13.59	65.05%	−3.88
**P6**	**66.99%**	52.43%	32.04%	59.22%	−7.77	**66.99%**	0.00
**P7**	50.49%	48.54%	37.86%	46.60%	−1.95	**52.43%**	+1.94
**P8**	**41.82%**	30.91%	17.27%	31.82%	−10.00	35.45%	−6.37
**P9**	52.73%	50.91%	30.91%	50.91%	−1.82	**54.55%**	+1.82
**P10**	**64.55%**	54.55%	35.45%	60.91%	−3.64	61.82%	−2.73
**P11**	56.36%	58.18%	35.45%	55.45%	+1.82	**60.00%**	+1.82
**P12**	45.45%	47.27%	35.45%	48.18%	+2.73	**51.82%**	+3.64
**P13**	**61.82%**	44.55%	34.55%	46.36%	−15.45	59.09%	−2.73
**P14**	64.55%	44.55%	34.55%	63.64%	−0.91	**66.36%**	+1.82
**P15**	62.73%	60.91%	36.36%	59.09%	−1.82	**63.64%**	+0.91
**P16**	59.09%	56.36%	32.73%	55.45%	−2.73	**60.91%**	+1.82
**P17**	53.64%	43.64%	30.91%	50.00%	−3.64	**54.55%**	+0.91
***Mean***	58.92%	51.43%	32.91%	54.62%	−3.74	**60.95%**	+1.48
***SD***	8.30%	7.75%	4.45%	8.77%	5.28	9.33%	4.70

Finally, we selected one of the classifiers as the meta classifier (SVM) for both stacking and CSS stacking classification methods and presented the results obtained per actor in Table 6 and the mean and SD values at the end. The results prove that using the CSS stacking classification method, the recognition accuracy is outperformed for all of the actors, except for actor P1, in which no improvements are appreciated. The improvements using the CSS stacking classification method range from 3.88 to 24.27 percentage points, with an average improvement of 10.06 points.

**Table 6:** First phase. Accuracies and improvements per person in percentage points comparing stacking and CSS stacking with EDA classification methods using SVM as the meta classifier. Mean and SD rows denote the average and standard deviation for each classification method and improvements considering all of the actors.

	Stacking	CSS Stacking	Improvements
**P1**	73.79%	73.79%	0.00
**P2**	52.43%	73.79%	+21.36
**P3**	49.51%	73.79%	+24.27
**P4**	44.66%	61.17%	+16.50
**P5**	55.34%	65.05%	+9.71
**P6**	55.34%	59.22%	+3.88
**P7**	40.78%	52.43%	+11.65
**P8**	24.55%	32.73%	+8.18
**P9**	46.36%	54.55%	+8.18
**P10**	55.45%	60.91%	+5.45
**P11**	52.73%	60.00%	+7.27
**P12**	43.64%	51.82%	+8.18
**P13**	40.91%	59.09%	+18.18
**P14**	60.00%	66.36%	+6.36
**P15**	59.09%	63.64%	+4.55
**P16**	48.18%	60.91%	+12.73
**P17**	50.00%	54.55%	+4,55
***Mean***	50.16%	60.22%	+10.06
***SD***	10.14	9.64	6.42

#### Statistical Tests

According to [73], we employed the Iman and Davenport test to detect statistical differences among the different classification paradigms. This test rejects the null hypothesis of equivalence between algorithms, since the *p*-value (0.000216) is lower than the *α*-value (0.1). Thus, Shaffer *post hoc* test is applied in order to find out which algorithms are distinctive among them. Table 7 shows the statistical differences obtained. As can be seen, the new approach statistically outperforms the results obtained with the standard multi-classifier systems (*p*-value <0.01). It is worth mentioning that there were no significant differences between CSS stacking and the best single paradigm. This is indeed due to the selection phase of the best approach among all of the single approaches used, before applying meta-classification, as explained in Section 4 of this paper.

**Table 7:** First phase. *p*-values of the pair-wise comparison between CSS stacking and the other multi-classifiers.

Hypothesis	Adjusted *p*
*CSS Stacking vs. Boosting*	**1.2094622076166072E-10**
*CSS Stacking vs. Bagging*	**2.2567292727265824E-4**
*CSS Stacking vs. Stacking*	**0.004635715398394891**

If the comparison is done pair-wise, the new approach shows better accuracy than each of the single classifiers used. For instance, comparing the SVM single classifier (the best one) with the new approach obtained using SVM as the meta classifier, the new paradigm outperforms the single one in 11 up to 17 actors.

### 6.2. Second Phase

Table 8 presents the results obtained by the CSS stacking classification method during the second phase, in which eGeMAPS parameters and a new combination of base classifiers in the first layer were employed for classification. Besides, a comparison with the CSS stacking built in the first phase and the corresponding improvements achieved are also presented. Both CSS stacking classifiers were constructed using the SVM as the meta-classifier, as it was the best meta-classifier in the first phase. As can be seen, the integration in the first layer of new base classifiers that performed well as single classifiers (especially the MLP classifier) and the employment of the eGeMAPS acoustic parameters, which also demonstrated their efficiency when comparing the results of single classifiers in both phases, helped improve the results for most actors. The most appreciable improvements are given by the actors P13, P12 and P8, which outperformed the previous results in the first phase by 20.70, 20.26 and 16.62 percentage points, respectively. In global terms, the average accuracy of the CSS stacking classifiers of the second phase outperformed the mean accuracy of the first phase by 4.56 percentage points, which demonstrated the effectiveness of the eGeMAPS parameters and the new classifiers included in the first layer of the CSS stacking classifiers of the second phase.

**Table 8:** Second phase. Accuracy percentages per actor for the CSS stacking classifier systems of the second phase (CSS stacking 2nd_Phase) and the comparison with the CSS stacking classifiers of the first phase (CSS stacking 1st_Phase). Mean and SD rows denote the average and standard deviation for each classifier for all of the actors.

	CSS Stacking 2nd_Phase	CSS Stacking 1st_Phase	Differences
**P1**	85.06%	73.79%	+11.27
**P2**	69.48%	73.79%	−4.31
**P3**	75.32%	73.79%	+1.53
**P4**	70.78%	61.17%	+9.61
**P5**	77.27%	65.05%	+12.22
**P6**	64.29%	59.22%	+5.07
**P7**	47.4%	52.43%	−5.03
**P8**	49.35%	32.73%	+16.62
**P9**	46.01%	54.55%	−8.54
**P10**	73.38%	60.91%	+12.47
**P11**	66.88%	60.00%	+6.88
**P12**	72.08%	51.82%	+20.26
**P13**	79.87%	59.09%	+20.78
**P14**	61.69%	66.36%	−4.67
**P15**	59.09%	63.64%	−4.55
**P16**	46.1%	60.91%	−14.81
**P17**	57.14%	54.55%	+2.59
*Mean*	64.78%	60.22%	+4.56
*SD*	12.34	9.94	10.46

In Appendix A, the confusion matrices scored by the CSS stacking classifiers in the second phase are presented for all of the actors.

### 6.3. Third Phase

In the third phase, ten classifiers were built for each of the classification systems (single, standard stacking and CSS stacking) employed on the Emo-DB. In Table 9, the results of the three best classifiers of each system are shown. The best result of the three classification systems is highlighted in bold per actor. Interestingly, MLP, RandomF and SVM are the best three classifiers for each of the classification systems.

Looking at the results, only for the A5 and A9 actors, the single classifier (RandomF) system scored the best accuracies; 80.00% and 82.14%, respectively, whilst the standard stacking classifiers achieved the worst results. However, the CSS stacking systems outperformed the results of single and standard stacking classifiers for the rest of the actors. The best result is achieved by the A2 actor, which scored an accuracy of 96.55% when the SVM acted as the meta-classifier. On average, the CSS stacking classifier with the SVM acting as the meta-classifier reached higher results, obtaining a mean of 82.45% accuracy for all of the actors. Considering that the human perception rate for the Emo-DB was set to 84% [43], this mean value of 82.45% can be seen as a promising result. Moreover, this score outperforms the results of other works in the literature over the Emo-DB, like the scores obtained in [43,74], which reached accuracies of 79% and 77%, respectively, although these works analyzed the whole database and used different machine learning algorithms and audio features. The overall results demonstrate the good performance of the CSS stacking classification paradigm and confirms the robustness of this classification system to deal with the emotion recognition in speech over several conditions and datasets.

**Table 9:** Third phase. Accuracy percentages per actor for the best three classifiers of each system built on the Berlin Emotional Speech database (Emo-DB). Mean and SD rows represent the average and standard deviation considering all of the actors.

	Single			Standard Stacking			CSS Stacking		
	MLP	RandomF	SVM	MLP	RandomF	SVM	MLP	RandomF	SVM
**A1**	79.59%	73.46%	77.55%	63.26%	71.42%	61.22%	79.59%	**81.63%**	79.59%
**A2**	94.82%	87.93%	86.20%	79.31%	89.65%	72.41%	93.10%	94.83%	**96.55%**
**A3**	74.41%	62.79%	67.44%	62.79%	67.44%	62.79%	74.42%	74.42%	**76.74%**
**A4**	84.21%	84.21%	81.57%	68.42%	71.05%	68.42%	**89.47%**	84.21%	86.84%
**A5**	63.63%	**80.00%**	72.72%	56.36%	65.45%	54.54%	67.27%	72.73%	78.18%
**A6**	77.14%	74.28%	80.00%	71.42%	68.57%	68.57%	**82.86%**	**82.86%**	**82.86%**
**A7**	78.68%	75.40%	72.13%	67.21%	70.49%	65.57%	77.05%	**80.33%**	78.69%
**A8**	78.26%	75.36%	78.26%	73.91%	76.81%	78.26%	82.61%	**86.96%**	85.51%
**A9**	67.85%	**82.14**%	66.07%	69.64%	71.42%	64.28%	76.79%	75.00%	75.00%
**A10**	74.64%	83.09%	76.05%	73.23%	71.83%	76.05%	83.10%	80.28%	**84.51%**
*Mean*	77.32%	77.87%	75.80%	68.55%	72.41%	67.21%	80.63%	81.32%	**82.45%**
*SD*	8.52	7.17	6.28	6.56	6.76	7.13	7.41	6.57	6.33

In Appendix A, the confusion matrices scored by the CSS stacking system with the SVM classifier acting as the meta-classifier are presented for all of the actors.

## 7. Conclusions and Future Work

Enabling computers the ability to recognize human emotions is an emergent research area. Continuing the authors’ previous work on the topic, in this article, different classification approaches have been presented and compared for the speech emotion recognition task. The experimentation was divided into three main phases, which differ from each other in: (1) the speech parametrization; (2) the base classifiers used to construct the classification systems; and (3) the dataset employed. The experiments were performed over the RekEmozio and Emo-DB datasets, which contain audio recordings in Basque, Spanish and German from several actors. As the emotional annotation in both datasets was performed using categories, the statistical approach was also turned into a categorical classification problem.

In the first phase, 10 single classifiers, 12 multi-classifiers (bagging, boosting and standard stacking generalization) and 10 final CSS stacking classifiers with the EDA classification method were built, evaluated and compared to each other. For single classifiers, the SVM became the best classifier among the ten algorithms employed, as it obtained the best accuracy for 13 of the 17 actors. If we focus on the performance of multi-classifiers, in most cases, they did not achieve better results compared to single classifiers. In addition, it is noticeable that although bagging was the classifier that reached the best results in most cases, it performed better only for seven of the 17 actors. The best accuracies for multi-classifiers ranged between 31.82% and 73.79%.

In comparison, the CSS stacking multi-classifier with EDA achieved higher accuracies than the single and multi-classifiers in most cases. Table 5 shows that, except for four out of 17 actors, CSS stacking with EDA outperformed the results of all of the other single and multi-classifiers tested in the first phase of this work. Furthermore, these results were statistically significant when comparing pair-wise with the other multi-classifiers. Therefore, it can be concluded from this first phase that multi-classifiers based on the CSS stacking method with EDA are a promising approach for emotion recognition in speech.

With regard to the second phase, a new parametrization based on the eGeMAPS acoustic parameters in addition to new base classifiers was employed to construct new CSS stacking classifiers using the best meta-classifier of the first phase. These new CSS stacking classifiers were compared to the CSS stacking classifiers from the first phase, in order to evaluate the impact of the new parameters and base classifiers included. The results from Table 8 concluded that the new configuration of the CSS stacking classifiers of the second phase outperformed the results obtained in the first phase in most cases. This demonstrated the good performance of the acoustic parameters and the new base classifiers employed in the second phase.

Finally, the third phase was focused on constructing single, standard stacking and CSS stacking classifiers for each of the actors in the well-known and freely-available Emo-DB. The results confirmed the good performance of the CSS stacking classifier system, which improved the accuracies obtained by the other classification systems for all actors, except two.

A future work for this research will be to perform new experiments on different databases, such as the Belfast naturalistic emotion database [10], the Vera am Mittag German audio-visual emotional speech database [75] and the FAUAibo Emotion Corpus [76], which include spontaneous speech, and the Berlin Database of Emotional Speech [12] and EMOVO[77] databases, in order to test out the efficiency of the presented new classification paradigm in other dataset conditions and domains. Besides, new standard classifiers will be explored, and a combination of data from several databases will be used with the aim of building speaker- and language-independent classification systems.

## Appendix

### Confusion Matrices for the CSS Stacking Classification Method of the Second and Third Phases

In this Appendix, one table per actor is presented, in which the confusion matrices obtained by the CSS stacking classifiers of the second and third phases are detailed. First, the confusion matrices from the RekEmozio database are shown from Table A1 to Table A17. Results of the Emo-DB are then presented from Table A18 to Table A27.

**Table A1:** P1 actor confusion matrix from the RekEmozio dataset in the second phase.

	Sadness	Fear	Joy	Anger	Surprise	Disgust	Neutral
Sadness	19	2	0	0	0	1	0
Fear	0	20	0	0	0	1	1
Joy	0	0	18	3	1	0	0
Anger	1	0	2	17	1	1	0
Surprise	0	0	3	2	17	0	0
Disgust	0	0	0	2	0	20	0
Neutral	1	0	0	0	0	1	20

**Table A2:** P2 actor confusion matrix from the RekEmozio dataset in the second phase.

	Sadness	Fear	Joy	Anger	Surprise	Disgust	Neutral
Sadness	16	2	0	0	0	1	3
Fear	1	20	0	0	0	1	0
Joy	0	0	17	2	3	0	0
Anger	0	0	3	19	0	0	0
Surprise	0	2	7	0	13	0	0
Disgust	2	1	2	0	3	5	9
Neutral	0	1	0	0	1	8	12

**Table A3:** P3 actor confusion matrix from the RekEmozio dataset in the second phase.

	Sadness	Fear	Joy	Anger	Surprise	Disgust	Neutral
Sadness	18	2	0	0	0	1	1
Fear	1	18	0	0	2	1	0
Joy	0	0	10	8	4	0	0
Anger	0	0	8	14	0	0	0
Surprise	0	1	2	2	16	1	0
Disgust	1	2	0	0	0	16	3
Neutral	3	0	0	0	0	1	18

**Table A4:** P4 actor confusion matrix from the RekEmozio dataset in the second phase.

	Sadness	Fear	Joy	Anger	Surprise	Disgust	Neutral
Sadness	21	0	0	0	0	0	1
Fear	0	16	5	0	0	1	0
Joy	0	4	12	4	2	0	0
Anger	0	1	4	13	2	0	2
Surprise	0	2	2	1	14	2	1
Disgust	0	2	1	2	0	15	2
Neutral	2	0	0	1	0	1	18

**Table A5:** P5 actor confusion matrix from the RekEmozio dataset in the second phase.

	Sadness	Fear	Joy	Anger	Surprise	Disgust	Neutral
Sadness	21	0	0	0	0	0	1
Fear	0	15	2	2	1	1	1
Joy	0	1	13	5	1	2	0
Anger	0	2	2	17	1	0	0
Surprise	0	0	1	5	16	0	0
Disgust	1	2	2	0	0	17	0
Neutral	1	0	0	0	0	1	20

**Table A6:** P6 actor confusion matrix from the RekEmozio dataset in the second phase.

	Sadness	Fear	Joy	Anger	Surprise	Disgust	Neutral
Sadness	20	0	1	0	1	0	0
Fear	0	16	0	1	3	2	0
Joy	2	0	16	1	0	1	2
Anger	2	5	3	8	3	1	0
Surprise	0	6	0	0	14	2	0
Disgust	3	2	2	3	0	9	3
Neutral	5	0	3	0	1	0	13

**Table A7:** P7 actor confusion matrix from the RekEmozio dataset in the second phase.

	Sadness	Fear	Joy	Anger	Surprise	Disgust	Neutral
Sadness	10	3	0	0	3	4	2
Fear	4	8	0	3	3	4	0
Joy	1	5	10	1	2	1	2
Anger	1	2	7	8	1	2	1
Surprise	2	4	1	0	12	2	1
Disgust	6	0	0	2	3	11	0
Neutral	4	1	1	2	0	0	14

**Table A8:** P8 actor confusion matrix from the RekEmozio dataset in the second phase.

	Sadness	Fear	Joy	Anger	Surprise	Disgust	Neutral
Sadness	12	5	0	0	0	2	3
Fear	5	8	1	0	0	6	2
Joy	1	1	10	5	4	0	1
Anger	0	1	5	11	5	0	0
Surprise	0	1	8	4	9	0	0
Disgust	3	7	0	0	0	11	1
Neutral	2	3	0	0	0	2	15

**Table A9:** P9 actor confusion matrix from the RekEmozio dataset in the second phase.

	Sadness	Fear	Joy	Anger	Surprise	Disgust	Neutral
Sadness	12	5	1	0	0	4	0
Fear	5	5	1	0	0	10	1
Joy	0	3	6	7	4	1	1
Anger	0	1	4	7	8	0	2
Surprise	1	1	4	5	11	0	0
Disgust	2	8	1	0	0	11	0
Neutral	0	2	0	2	0	2	16

**Table A10:** P10 actor confusion matrix from the RekEmozio dataset in the second phase.

	Sadness	Fear	Joy	Anger	Surprise	Disgust	Neutral
Sadness	19	0	0	0	0	1	2
Fear	0	16	1	0	1	4	0
Joy	0	1	12	4	1	4	0
Anger	0	1	3	16	0	2	0
Surprise	1	1	1	0	19	0	0
Disgust	0	4	3	4	1	10	0
Neutral	0	0	0	0	0	1	21

**Table A11:** P11 actor confusion matrix from the RekEmozio dataset in the second phase.

	Sadness	Fear	Joy	Anger	Surprise	Disgust	Neutral
Sadness	20	0	0	1	0	0	1
Fear	0	14	3	1	2	2	0
Joy	0	2	14	4	2	0	0
Anger	2	0	4	13	1	2	0
Surprise	0	3	3	0	11	4	1
Disgust	0	1	2	5	2	12	0
Neutral	2	0	0	0	0	1	19

**Table A12:** P12 actor confusion matrix from the RekEmozio dataset in the second phase.

	Sadness	Fear	Joy	Anger	Surprise	Disgust	Neutral
Sadness	20	0	0	0	0	1	1
Fear	1	13	1	0	0	6	1
Joy	0	1	18	2	1	0	0
Anger	0	1	2	16	2	1	0
Surprise	0	0	2	3	17	0	0
Disgust	2	3	0	1	1	11	4
Neutral	1	0	0	0	1	4	16

**Table A13:** P13 actor confusion matrix from the RekEmozio dataset in the second phase.

	Sadness	Fear	Joy	Anger	Surprise	Disgust	Neutral
Sadness	20	1	0	0	0	1	0
Fear	2	17	1	1	1	0	0
Joy	0	1	18	1	0	0	2
Anger	0	0	1	20	0	0	1
Surprise	0	3	1	1	17	0	0
Disgust	1	0	1	0	1	15	4
Neutral	0	1	1	0	1	3	16

**Table A14:** P14 actor confusion matrix from the RekEmozio dataset in the second phase.

	Sadness	Fear	Joy	Anger	Surprise	Disgust	Neutral
Sadness	7	1	0	0	0	7	7
Fear	2	14	1	0	2	3	0
Joy	0	1	14	2	5	0	0
Anger	0	0	2	17	2	0	1
Surprise	0	2	10	3	7	0	0
Disgust	5	2	0	0	0	14	1
Neutral	2	0	0	0	0	1	19

**Table A15:** P15 actor confusion matrix from the RekEmozio dataset in the second phase.

	Sadness	Fear	Joy	Anger	Surprise	Disgust	Neutral
Sadness	10	0	0	0	0	5	7
Fear	3	14	1	1	1	1	1
Joy	0	0	16	3	3	0	0
Anger	0	1	4	8	6	2	1
Surprise	0	0	5	3	13	1	0
Disgust	4	4	0	0	0	13	1
Neutral	4	0	0	0	0	1	17

**Table A16:** P16 actor confusion matrix from the RekEmozio dataset in the second phase.

	Sadness	Fear	Joy	Anger	Surprise	Disgust	Neutral
Sadness	16	0	0	0	0	4	2
Fear	0	9	5	4	2	0	2
Joy	1	3	7	3	4	3	1
Anger	2	4	3	8	4	1	0
Surprise	0	4	2	3	13	0	0
Disgust	4	0	0	0	2	12	4
Neutral	1	3	2	0	0	3	13

**Table A17:** P17 actor confusion matrix from the RekEmozio dataset in the second phase.

	Sadness	Fear	Joy	Anger	Surprise	Disgust	Neutral
Sadness	7	1	0	0	0	8	6
Fear	1	9	2	3	4	3	0
Joy	0	2	7	9	3	1	0
Anger	0	6	7	4	5	0	0
Surprise	0	2	3	2	10	5	0
Disgust	3	3	0	1	5	9	1
Neutral	8	0	1	0	0	2	11

**Table A18:** A1 actor confusion matrix from the Emo-DB in the third phase.

	Sadness	Fear	Joy	Anger	Surprise	Disgust	Neutral
Sadness	13	0	0	1	0	0	0
Fear	0	2	0	0	0	0	3
Joy	0	0	0	0	1	0	0
Anger	3	0	0	1	0	0	0
Surprise	0	0	0	0	7	0	0
Disgust	0	0	0	0	0	7	0
Neutral	0	2	0	0	0	0	9

**Table A19:** A2 actor confusion matrix from the Emo-DB in the third phase.

	Sadness	Fear	Joy	Anger	Surprise	Disgust	Neutral
Sadness	11	0	0	0	1	0	0
Fear	0	10	0	0	0	0	0
Joy	0	0	0	0	0	0	0
Anger	0	0	0	6	0	0	0
Surprise	0	0	0	0	11	0	0
Disgust	0	0	0	0	0	9	0
Neutral	0	1	0	0	0	0	9

**Table A20:** A3 actor confusion matrix from the Emo-DB in the third phase.

	Sadness	Fear	Joy	Anger	Surprise	Disgust	Neutral
Sadness	12	0	1	0	0	0	0
Fear	0	0	0	0	0	0	4
Joy	1	0	7	0	0	0	0
Anger	1	0	0	0	0	0	0
Surprise	2	0	0	0	2	0	0
Disgust	0	0	0	0	0	4	0
Neutral	0	1	0	0	0	0	8

**Table A21:** A4 actor confusion matrix from the Emo-DB in the third phase.

	Sadness	Fear	Joy	Anger	Surprise	Disgust	Neutral
Sadness	10	0	0	0	0	0	0
Fear	0	7	0	0	0	1	0
Joy	0	0	0	1	0	0	0
Anger	0	0	0	7	1	0	0
Surprise	0	0	0	0	4	0	0
Disgust	0	0	0	0	0	3	0
Neutral	0	2	0	0	0	0	2

**Table A22:** A5 actor confusion matrix from the Emo-DB in the third phase.

	Sadness	Fear	Joy	Anger	Surprise	Disgust	Neutral
Sadness	8	0	0	1	2	0	0
Fear	0	4	0	0	0	3	1
Joy	0	0	0	2	0	0	0
Anger	0	0	0	10	0	0	0
Surprise	2	0	0	1	5	0	0
Disgust	0	0	0	0	0	7	0
Neutral	0	0	0	0	0	0	9

**Table A23:** A6 actor confusion matrix from the Emo-DB in the third phase.

	Sadness	Fear	Joy	Anger	Surprise	Disgust	Neutral
Sadness	12	0	0	0	0	0	0
Fear	0	3	0	0	2	0	0
Joy	0	0	1	1	0	0	0
Anger	0	1	0	5	0	0	0
Surprise	1	1	0	0	0	0	0
Disgust	0	0	0	0	0	4	0
Neutral	0	0	0	0	0	0	4

**Table A24:** A7 actor confusion matrix from the Emo-DB in the third phase.

	Sadness	Fear	Joy	Anger	Surprise	Disgust	Neutral
Sadness	11	0	0	0	1	0	0
Fear	0	9	0	0	0	0	1
Joy	0	1	6	0	0	0	1
Anger	1	0	1	5	0	0	0
Surprise	1	0	0	0	9	0	0
Disgust	0	0	0	0	0	5	0
Neutral	0	6	0	0	0	0	3

**Table A25:** A8 actor confusion matrix from the Emo-DB in the third phase.

	Sadness	Fear	Joy	Anger	Surprise	Disgust	Neutral
Sadness	16	0	0	0	0	0	0
Fear	0	7	0	0	0	0	1
Joy	0	0	7	1	0	0	0
Anger	0	0	1	10	1	0	0
Surprise	6	0	0	0	2	0	0
Disgust	0	0	0	0	0	10	0
Neutral	0	0	0	0	0	0	7

**Table A26:** A9 actor confusion matrix from the Emo-DB in the third phase.

	Sadness	Fear	Joy	Anger	Surprise	Disgust	Neutral
Sadness	11	0	0	1	1	0	0
Fear	0	7	0	0	0	1	1
Joy	0	0	4	0	0	0	1
Anger	2	0	0	6	0	0	0
Surprise	2	0	0	2	2	0	0
Disgust	0	0	0	0	0	4	0
Neutral	0	2	0	0	0	1	8

**Table A27:** A10 actor confusion matrix from the Emo-DB in the third phase.

	Sadness	Fear	Joy	Anger	Surprise	Disgust	Neutral
Sadness	12	0	0	1	1	0	0
Fear	0	12	1	0	0	0	1
Joy	0	1	10	0	0	0	0
Anger	1	0	1	4	1	0	0
Surprise	1	0	0	0	10	0	0
Disgust	0	0	0	0	0	9	0
Neutral	0	2	0	0	0	0	3
